# Placenta-Derived Exosomes as a Modulator in Maternal Immune Tolerance During Pregnancy

**DOI:** 10.3389/fimmu.2021.671093

**Published:** 2021-05-11

**Authors:** Kunfeng Bai, Xintong Li, Jiangming Zhong, Ernest H. Y. Ng, William S.B. Yeung, Cheuk-Lun Lee, Philip C. N. Chiu

**Affiliations:** ^1^ Department of Obstetrics and Gynaecology, Li Ka Shing (LKS) Faculty of Medicine, The University of Hong Kong, Hong Kong, Hong Kong; ^2^ The University of Hong Kong Shenzhen Key Laboratory of Fertility Regulation, The University of Hong Kong-Shenzhen Hospital, Shenzhen, China

**Keywords:** placenta, exosomes, maternal immune tolerance, preeclampsia, gestational diabetes mellitus, preterm

## Abstract

Exosomes are a subset of extracellular vesicles with an average diameter of ~100nm. Exosomes are released by all cells through an endosome-dependent pathway and carry nucleic acids, proteins, lipids, cytokines and metabolites, mirroring the state of the originating cells. The function of exosomes has been implicated in various reproduction processes, such as embryo development, implantation, decidualization and placentation. Placenta-derived exosomes (pEXO) can be detected in the maternal blood as early as 6 weeks after conception and their levels increase with gestational age. Importantly, alternations in the molecular signatures of pEXO are observed in pregnancy-related complications. Thus, these differentially expressed molecules could be the potential biomarkers for diagnosis of the pregnancy-associated diseases. Recent studies have demonstrated that pEXO play a key role in the establishment of maternal immune tolerance, which is critical for a successful pregnancy. To gain a better understanding of the underlying mechanism, we highlighted the advanced studies of pEXO on immune cells in pregnancy.

## Introduction

Pregnancy is a complex process associated with numerous biological changes in the maternal body and our understanding of the complicated relationship between the mother and its semi-allograft fetus is still limited (1). An immune tolerant environment is a prerequisite to a successful pregnancy. However, the understanding of how the fetus avoids maternal immune rejection is an enigma. During pregnancy, the mother needs to have a competent immune system against infection but is tolerant to the developing fetus. Any disruption of the immune tolerance would lead to adverse pregnancy outcomes such as recurrent pregnancy loss (2), miscarriage (3) and preeclampsia (4).

The maternal immune system undergoes a wide variety of biological changes during pregnancy. These include decidual immune cell mobilization, re-distribution and polarization at a local level (5–7) and a universal immunosuppressive state at a systemic level (8, 9). In humans, the trophectoderm of blastocyst protects the growing embryo at implantation (10). After implantation, the syncytiotrophoblast (STB) derived from the trophectoderm, surrounds most of the chorionic villi, and prevents the fetus from a direct contract with the maternal blood. The trophoblasts have a unique human leukocyte antigen (HLA) profile (11). For example, the STBs are HLA null and are considered as immunologically neutral, while the extravillous trophoblast cells (EVTs) express an unusual repertoire of HLA-I molecules including HLA-G, HLA-C and HLA-E (12, 13). Furthermore, the STBs produce various immunoregulatory factors such as interleukin 10 (IL10) (14), macrophage colony-stimulating factor (M-CSF) (15) and IL-35 (16), which contribute to maternal immune tolerance as well.

Exosomes, firstly regarded as cell burden, are involved in the process of antigen presentation, signal transduction and immune responses. Placenta STB has been demonstrated to continuously releases extracellular vesicles (EVs), microvesicles and exosomes, to the maternal circulation (17, 18). The study on pEXO can date back to 1999 (19) and our understanding of pEXO are significantly increased due to advances in technologies of exosome purification in the last decade. Beyond that, exosomes from other sources-such as stem cells and tumor-have a critical role in growth, metabolism and development. The function of pEXO has been implicated in conferring viral resistance to non-placenta cells, inhibiting T cells recognition and activation, and promoting macrophage differentiation and polarization during pregnancy. Here, we summarize the current knowledge of pEXO in the establishment of maternal immune tolerance and outlined an overview role of its application in disease diagnosis.

## Placenta-Derived Extracellular Vesicles

### Extracellular Vesicles

Communication among our body cells is traditionally considered to be through autocrine, paracrine, endocrine and direct cell-cell contact. Other than that, EVs are another means of cell-cell communication. According to the guidelines of the International Society for Extracellular Vesicles (ISEV), EVs are lipid-bound vesicles with a diameter ranging from 30 nm to 2 μm released from all kinds of cells (20, 21). Based on the biogenesis process, EVs generally fall into two categories, ectosomes and exosomes (**Table 1**) (20). Ectosomes are vesicles produced by cells *via* direct outward budding. They can be further divided into microvesicles (MVs, 200 nm ~ 1 μm in diameter) and apoptotic bodies (APs, 1 μm ~ 5 μm in diameter) (22). By contrast, exosomes are nano-sized particles with a size ranging from 30 nm to 200 nm in diameter (100 nm on average) generated by inward budding of the plasma membrane *via* a multi-vesicular system (21).

**Table 1:** Summary of different subtypes of placenta-derived extracellular vesicles.

	Exosomes	Microvesicles	Apoptotic bodies	Syncytial nuclear aggregates (SNA)
**Size**	30nm ~ 200nm	200nm ~ 1μm	1μm ~ 5μm	20μ-200μm
**Origin**	Endocytic pathway	Plasma membrane	Plasma membrane	Syncytiotrophoblast
**Function**	Intercellular communication	Intercellular communication	Facilitate phagocytosis	Unclear
**Contents**	Proteins, miRNA, mRNA, lipid and metabolites	Proteins, miRNA, mRNA, lipid and metabolites	Nuclear fractions, cellular organelles	Nucleus, proteins, miRNA, mRNA, lipid, metabolites
**Markers**	Alix, CD81, CD63, CD9	Integrins, selectins, CD40	Annexin V, phosphatidylserine	Nucleus cluster

Initially, EVs are considered as cell debris for the purpose of maintaining cellular homeostasis (23, 24). EVs carry various molecular cargoes, such as proteins, microRNAs (miRNAs), mRNAs, lipids and metabolites, which endow the EVs with capacity as a natural vehicle for intercellular communication (25). The function of exosomes has been well documented in tumorigenesis, metastasis, regeneration, mammalian reproduction and development (26). Certain miRNAs are enriched in exosomes compared to those in the cells of origin, indicating that the process of exosomes biogenesis is not random, but in a pre-primed manner (27, 28). However, the mechanism underlying the exosomal cargo incorporation is still unclear.

### Placenta Syncytial Nuclear Aggregates, Microvesicles and Exosomes

**Figure 1:**
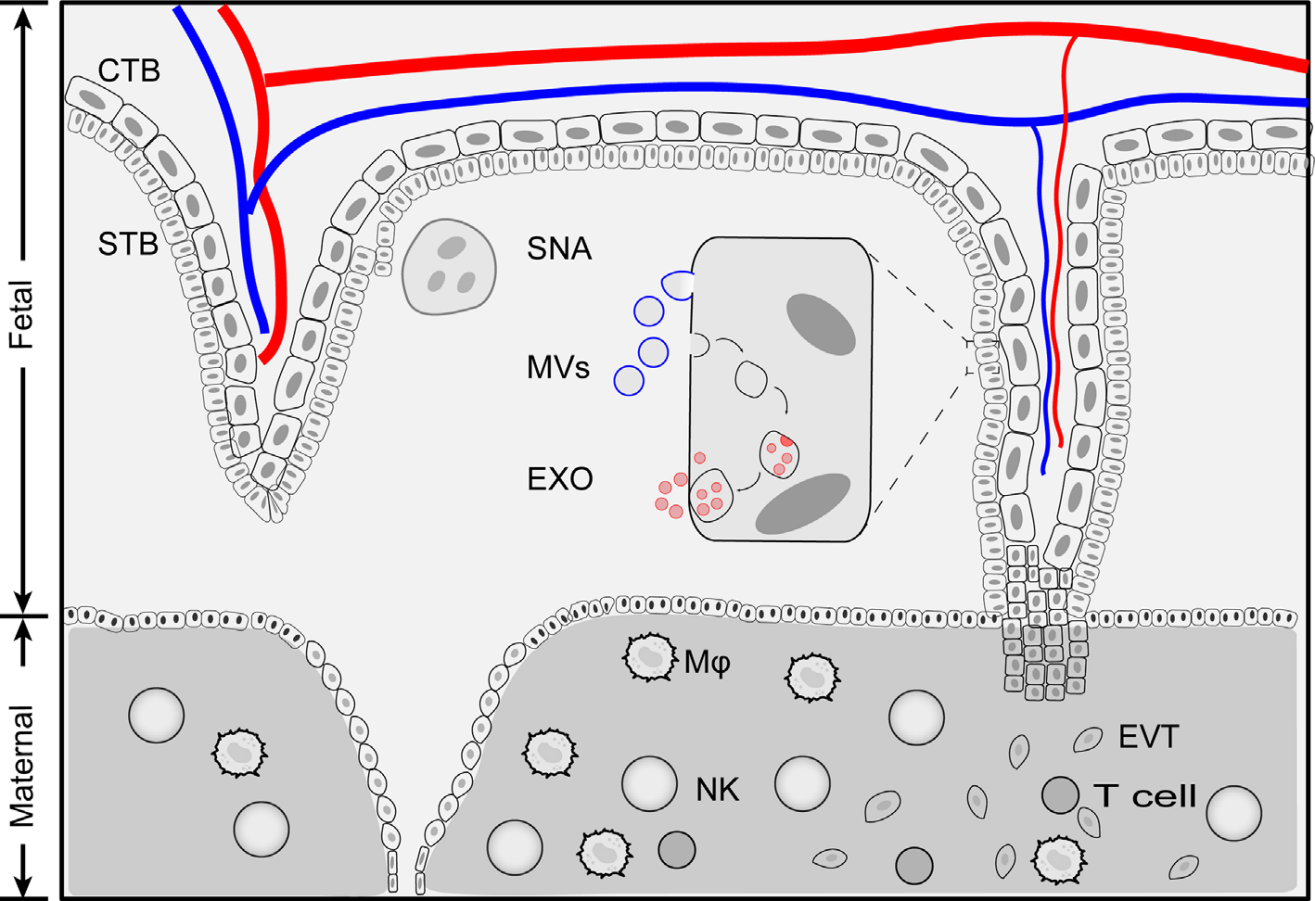
Schematic illustration of placenta extracellular vesicles. Placenta derived extracellular vesicles can be divided into four categories: exosomes, microvesicles, apoptotic bodies and syncytial nuclear aggregates based on size and biogenesis pathway. Exosomes are generated by multivesiculuar body (MVB)-intraluminal vesicles (ILVs) system. first MVBs are generated by plasma membrane inward budding. Further, invagination of the late endosomes forms intraluminal vesicles (exosomes) within multivesiculuar body (MVB). Exosomes release to extracellular space when MVB fuse with membrane plasma. During this processes, membrane components and cytosolic materials are loaded into exosomes. Microvesicles and apoptotic bodies are produced by outward budding of plasma membrane and the size range of 200 nm - 5 μm. Syncytial nuclear aggregates (SNA) are clusters of syncntiotrophoblast with multiple nuclei per SNA. CTB, cytotrophoblast; Exo, exosomes; EVT, extravillous trophoblast; MVs, microvesicles; Mφ, macrophage; NK, Natural killer cells; STB, syncytiotrophoblast; SNA, syncytial nuclear aggregates.

During pregnancy, the placenta actively releases EVs into the bloodstream of the mother. The STB is the major source of placenta-derived EVs in the maternal blood (29, 30). Unlike those EVs that originate from other tissues, placenta-derived EVs are divided into three categories based on their sizes: syncytial nuclear aggregates (SNAs), microvesicles (MVs) and exosomes (**Figure 1**) (31).

Placenta-derived SNAs, also known as syncytial knot, are the clusters of multinucleated aggregate of syncytial nuclei (20 μm~200 μm in diameter, averaged 60 nuclei per knot) extruded from STB (32). The formation of placenta-derived SNAs is generally considered as a degenerative process, an aging change and an indicator of trophoblastic state when exposed to ischemia or hypoxia (18, 31, 33). The history of placenta-derived SNAs can be dated back to 120 years ago when they were first found in the lungs of post-mortem women (34). However, the origin and formation of SNAs are far from clear. Nuclei within SNAs exhibited condensed morphology compared to the STB and showed little evidence of apoptosis, indicating that SNAs are not fragmented STB (31). SNAs could be used as an alternative source of fetal DNA for prenatal diagnosis (35). The levels of SNAs increase as gestation proceeds and are found to be correlated in pregnancy complications such as preeclampsia (36).

The biological function of placenta-derived MVs is broad, encompassing immune cell activation, proliferation, and endothelial hemostasis (37). MVs collected from normal placenta perfusion have a pro-inflammatory effect *via* activating monocytes and B cells (38). Inhibition of MV internalization cannot block placenta-derived MV-mediated activation of monocytes and B cells indicating that membrane-bound proteins are the key players of the phenomenon. Proteomic analysis revealed that the differential expressed proteins between MVs from normal pregnancy and preeclampsia patients are related to mitochondria, transmembrane transport and membrane transporter activity (39).

pEXO can interact with various target cells including endothelium, T cells, monocytes, natural killer (NK) cells and macrophages. pEXO are found to protect endothelial cells from viral infection (40), inhibit NK cytotoxicity (41), constrain T cell proliferation (42) and promote monocyte differentiation and macrophage polarization (43). During pregnancy, pEXO can be detected as early as 6 weeks (44) and their number increases gradually and finally peaks at term. Pathologically, the levels of exosomes have been correlated with pregnancy-associated complications such as preeclampsia (45), gestational diabetes mellitus (46) and preterm birth (47), which will be described later in this review. Interestingly, all these complications have been demonstrated to associated with alteration of immune system during pregnancy. However, the detailed roles and mechanisms of pEXO in maternal immune adaption and placental development are still obscure.

## pEXO Preparation and Isolation

To date, pEXO are mainly purified from four types of sources: maternal blood, placental perfusate, placental explant culture, and primary trophoblast culture. However, pEXO isolated by different methods have distinct effects on endothelial cells, T cells and other cells [Reviewed in (48–50)]. Generally, the yield of placenta exosomes in the maternal blood is relatively low. On the other hand, the yields of exosomes from placental perfusion and explant culture are relatively high but the purity of the isolated exosomes is a concern. Since differences in content and immunoregulatory activities of exosome from primary cell and its established cell lines have been reported (51–53). Primary cells are currently the best source of exosomes preparation when sample availability is adequate. However, exosomes from trophoblast cell lines with gene manipulation could also provide valuable information regarding trophoblast-specific gene expression and function (54, 55).

**Figure 2:**
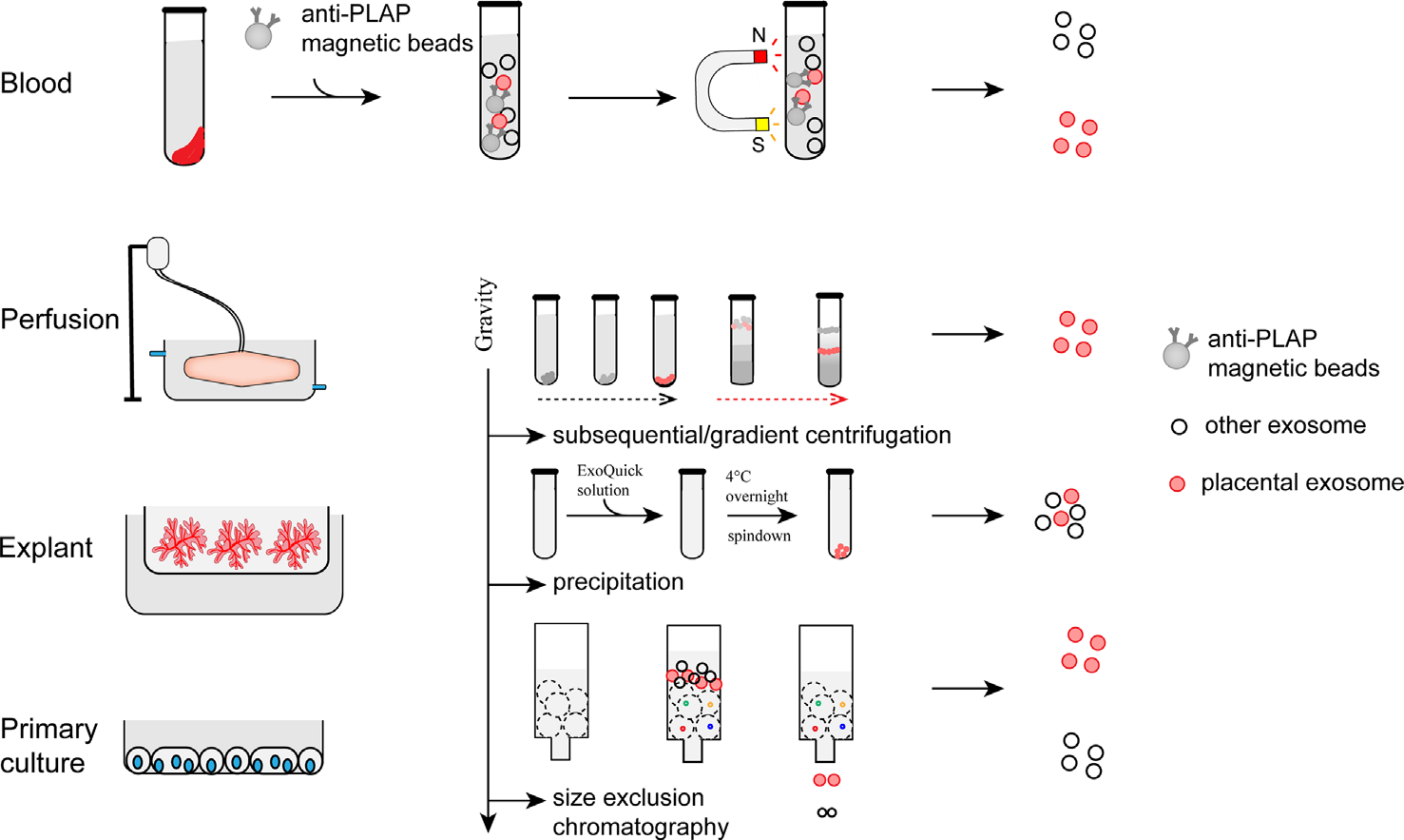
Preparation and isolation of pEXO. Exosomes extruded from placenta can be purified from blood plasma, medium of placental perfusion, explant culture and primary trophoblast culture through immune-capture, ultracentrifugation, gradient centrifugation, or size exclusion chromatography.

Immuno-capture, centrifugation, precipitation, and size exclusion chromatography are commonly used to isolate exosomes from the biological fluid or culture medium (21, 56–59) (**Figure 2**). The immuno-capture method is commonly used to isolate pEXO in plasma (60). Magnetic beads coated with monoclonal anti-PLAP (placental alkaline phosphatase) antibodies capture placenta-specific exosomes through antigen-antibody interaction. Ultracentrifugation and gradient ultracentrifugation are the most widely used methods in exosome studies. In these methods, EVs are isolated by differential centrifugal forces. Dead cells and cell debris are pelleted with a relatively low centrifugal force (300g for dead cells and 2000g for cellular debris). Higher centrifugal force at 16,500g is then applied to separate the MVs. Exosomes can be harvested by ultracentrifugation at >100,000g, for 60 minutes. To enhance the purity of exosomes, gradient ultracentrifugation is employed to separate different subtypes of exosomes (59). Precipitation is another method for exosome purification (61, 62). Polyethylene glycol (PEG) functions as a water-excluding molecule that precipitates the exosomes out of the aqueous phase. Usually, exosomes are isolated by a low-speed centrifugation after incubating the sample with a precipitation solution containing PEG. However, proteins may also be precipitated by PEG which could result in a lower purity than those generated by ultracentrifugation. Size exclusion chromatography (SEC) has also been used for exosome purification (63, 64). In this method, exosomes and soluble proteins are separated by a porous matrix. Exosomes that are larger than the size cutoff of the matrix are eluted faster than the soluble proteins. Compared to other methods, exosomes isolated by SEC have a higher purity but lower yields. However, all the methods have their limitation in terms of efficiency and purity. To bridge this gap, new technologies and standardization of protocols for pEXO isolation are needed in future studies.

## Maternal Adaptation of Immune System Response at Early Pregnancy

Placenta-driven immune tolerance is a hallmark of a successful pregnancy when exposed to fetal antigens (65–69). Paternal antigens encounter the maternal immune system when the placenta villi are in contact with the maternal blood and when the EVTs interact with the human decidua. Strikingly, the maternal immune cells are abundant in the human decidua in early pregnancy accounting for 40% of the total decidual cells. Among them, NK cells (70%) and macrophages (20%) are the two largest subpopulations, with the rest constituted by T cells. Dendritic cells and B cells are almost absent in the human decidua (70). Interestingly, endometrium exhibits a sharp increase in NK cells and macrophages and a steep decline in T cells during the secretory phase of menstrual cycle, indicating that hormones may influence immune cell population and functions. Although the total cell numbers of the decidual immune cells in the peri-implantation and post-implantation periods are similar, their phenotypes and functions are dramatically different (71, 72).

It is generally accepted that a T-helper type-2 (Th2) cytokine prevailing environment is important in pregnancy (73). The proportion of Th2 cytokines-secreting cells in the endometrium are significantly higher in pregnant women in the first trimester than in non-pregnant women (73–75). On the other hand, Th1 cytokine-dominated immune responses are associated with implantation failures (76), abortion (77) and preeclampsia (78). The excessive Th1 cytokines are also associated with an elevated number of activated CD8^+^ T cells (79), M1 macrophages (80), Th-17 cells (81) in the decidua. However, several Th1 cytokines such as interferon (IFN)-γ and tumor necrosis factor-α are important in uterine vascular remodeling (82) and implantation (83), suggesting that the Th-1/Th-2 paradigm for pregnancy may be too simplistic. Recently, the concept of Th-1/Th-2 paradigm was gradually expanded to Th1/Th2/Th17/Treg paradigm due to the discovery of new Th cell subsets at the maternal-fetal interface (84, 85).

Systemic changes in peripheral immune cells are also essential for a successful pregnancy. It is supported by the observations in immunodeficient mice (**Table 2**). In general, adaptive immune cell-deficient female mice are fertile, whereas innate immune cell-deficient female mice are often accompanied by a compromised reproductive performance. However, the mechanism responsible for this observation is still unknown. The significance of peripheral immune cells in pregnancy is also well manifested in pregnant mothers with rheumatoid arthritis, an autoimmune disease which was partially subsided during pregnancy (97). Peripheral Treg cells, granulocytes and monocytic myeloid-derived suppressor cells (M-MDSC) are significantly increased when compared to non-pregnant women (98–100). In contrast, the number of T cells and B cells remain stable (8, 101). Moreover, the cytotoxicity of peripheral NK cells from pregnant women is well constrained when compared to non-pregnant individuals (102, 103).

**Table 2:** Reproductive performance of immunodeficient mouse model.

	Immune cell deficiency							Innate immune cell depletion	
Cell types	Nude	SCID	Nod-SCID	Rag-/-	Rag-/- γc-/-	Treg-/-	Csf1-null	CD11b+	MDSC
Mature B cells	Present	Absent	Absent	Absent	Absent	Present	Present	Present	Present
Mature T cells	Absent	Absent	Absent	Absent	Absent	Present	Present	Present	Present
Dendritic cells	Present	Present	Defective	Present	Present	Present	Present	Present	Present
NK cells	Present	Present	Defective	Present	Present	Present	Present	Present	Present
Treg	N/A	N/A	N/A	N/A	N/A	Absent	Present	Present	Present
Macrophages	Present	Present	Present	Present	Present	Present	Absent	Absent	Absent
Monocytes	Present	Present	Present	Present	Present	Present	Defective	Absent	Defective
MDSC	N/A	N/A	N/A	N/A	N/A	Present	N/A	N/A	Absent
Reproductivity	Fertile	Fertile	Impaired	Fertile	Fertile	Impaired	Infertile	Infertile	Impaired
Reference	(86)	(87)	(88, 89)	(90)	(91)	(92)	(93, 94)	(95)	(96)

MDSC, myeloid-derived suppressor cells.

## pEXO as a Modulator of Maternal Immune Tolerance

Given that exosomes, but not other EVs, are generated through the endosomal pathway, biological molecules encompassed by the exosomes are believed to have specific functions in cell-cell crosstalk. The placenta secretes a large number of exosomes into the maternal circulation. The NK cells, macrophages and T-cells are the three largest cell types making up >90% of the immune cells at the fetal-maternal interface in the post-implantation period. Thus, this review focuses on the effect of pEXO on these three immune cell populations. Yet it should be emphasized that exosomes are also involved in mediating the bi-direction communications between endometrium and embryo during peri-implantation and implantation phase (104–106). For example, endometrial epithelial cell-derived exosomes promote embryo attachment during implantation *via* miR-30d-dependent upregulation of integrins or through activation of focal adhesion kinase (FAK) signaling pathway (107, 108). Another study shows that diapausing endometrial epithelial cell-derived exosomes enriched with miR-let-7 can protect the embryo from collapsing (109). Conversely, embryo-derived exosomes have been detected in spent embryo culture medium. These exosomes can be internalized by endometrial epithelial/stromal cells (110, 111) and promote endometrial receptivity (112–114).

### Natural Killer Cells

#### Peripheral Blood Natural Killer Cells

NK cells in peripheral blood are divided into two groups: over 90% of peripheral NK cells (pNK) are CD56^dim^ CD16^+^ which are cytotoxic cells; the rest are CD56^+^ CD16^-^ NK cells which are less cytotoxic and can migrate into peripheral tissues. Compare to non-pregnant, pNK of pregnant women have a higher expression of Tim-3 (115), galectin-1 (102) and lower secretion of IFN-γ (103). The cytotoxic activities of pNK at early pregnancy are controversial (101, 116). Moreover, overactivated pNK are associated with repeated implantation and unexplained spontaneous abortion (117). Currently, there is no report on the effect of pEXO on pNK.

#### Endometrial NK Cells (eNK) and Decidual NK Cells (dNK)

NK cells represent the largest fraction of lymphocytes in the endometrium during the late-secretory phase and early pregnancy. Unlike pNK, majority of the endometrium NK cells (eNK) are CD56^+^ CD16^-^ with a minority being cytotoxic CD56^dim^ CD16^+^. The transformation from eNK to decidual NK cells (dNK) occurs upon implantation, resulting in two cell subsets with distinct transcriptional profiles. Strikingly, the eNK are more active than the dNK as 70% of differentially expressed genes are highly expressed in the eNK (118). On the other hand, the eNK have no expression of NKp30 and cannot produce VEGF and placental growth factor (119). The phenotype and KIR repertoire are also different between the two type NKs; the dNK have a higher expression of KIR2D, the killer immunoglobulin-like receptor for HLA-C recognition than the circulating NK and the non-pregnant eNK (72, 120).

Decidual NK cells (dNK) are abundant in the maternal-fetal interface at early pregnancy- accounting for up to 70% of total lymphocytes in decidua (121). The number of dNK gradually increases upon embryo implantation, peaks at 8-10 weeks of gestation, and returns to the original level at term. In general, dNK have low cytotoxicity and are prone to produce more growth factors with immunomodulatory activities (122). Elevated dNK cytotoxicity is associated with recurrent spontaneous abortion due to increased lysis activity (123). This was, in part, mediated by the up-regulation of NKp44 and NKp46 cytotoxicity receptors on CD56^bright^ CD16^-^ and CD56^dim^CD16^+^ cells (124). In fetal/neonatal alloimmune thrombocytopenia (FNAIT), activated dNK with increased cytotoxicity induce trophoblast apoptosis (125). A recent single-cell study (126) classified the dNK into three subsets: dNK1 (CD39^+^KIR2DL^+^ITGB2^-^CD103^-^), dNK2 (CD39^-^KIR2DL^+^ITGB2^+^CD103^-^) and dNK3 (CD39^-^KIR2DL^-^ITGB2^+^). The origin of the dNK remains uncertain, though they are thought to be derived from the NK precursor in the endometrium, or recruited from the circulating NK (126) and/or renewed by the CD34^+^ progenitor cells (127).

dNK are localized closely to EVT and spiral artery (128). They are vital to various processes of pregnancy including embryo implantation, immunomodulation, trophoblast differentiation and invasion, and endothelial cell remodeling. dNK also express unique NK receptors (e.g. 2B4, KIR2DL, ILT2) for interaction with their corresponding ligands (e.g. HLA-C, -E, -G) on EVT to fine-tune their cytolytic activity (129) within the maternal-fetal interface during the first trimester of pregnancy. Recent studies also suggest novel properties of dNK such as providing osteoglycin (OGN) and osteopontin (OPT) for fetal development (130) and selectively killing the pathogenic bacteria inside the trophoblast by injection of granulysin through nanotubes (131).

#### Effect of pEXO on NK Cells

pEXO can be internalized by NK cells *in vivo* (132) and *in vitro* (**Supplementary Table 1**) (133), which mediate the crosstalk between the placenta and the maternal immune system. The cytotoxic activity of NK cells mainly attributes to its activating receptors on the plasma membrane. NK group 2 member D (NKG2D) is widely expressed on the NK cells, activated CD8^+^ T cells and macrophages for removal of infected cells or foreign pathogens. NKG2D is remarkably downregulated in NK cells by NKG2D ligands expressed on pEXO (**Supplementary Table 1**) (134).

NKp30 is another activating receptor on the NK cells responsible for eliminating cancer cells and inducing dendritic cells maturation by secretion of tumor necrosis factor-alpha (TNF-α), interferon-gamma (IFN-γ), perforins and granzymes (135). B7H6, one of NKp30 endogenous ligands, is widely expressed on cancer cells and trophoblasts, while soluble B7H6 (sB7H6) was a decoy agent for ligand-receptor interaction and compromising NK cytotoxicity. High levels of exosome-packed sB7H6 or soluble B7H6 are correlated with poor tumor prognosis, likely due to inhibition of the NK cytotoxicity against the tumor cells (136). During pregnancy, both exosome-packed B7H6 and sB7H6 are present in the serum of pregnant women (**Supplementary Table 1**), indicating its potential contribution *via* a similar mechanism to inhibit NK cells in the establishment of maternal immune tolerance (137).

In addition to reducing the cytotoxicity of NK cells, exosomes from the serum of pregnant women can selectively increase the caspase-3 activity in CD56^dim^ NK cells, pointing to an alternative way of exosome-mediated immune tolerance by inducing apoptosis of the CD56^dim^ NK cells (**Supplementary Table 1**) (133). pEXO proteomic study showed that glycodelin A (GdA), a glycoprotein with immunosuppressive activities, is abundantly expressed in human decidua and pEXO (138). We demonstrated that decidua-derived GdA stimulated the conversion of peripheral CD56^bright^ CD16^-^ NK cells to cells with a decidual NK cell-like phenotype *via* upregulation of CD9, CD49a and production of VEGF (139). Together, this evidence indicated that the pEXO contribute to maternal immune tolerance through modulating NK cytotoxicity, inducing CD56^dim^ NK cells apoptosis and promoting the development of decidual NK cell-like phenotype.

### Monocytes and Macrophages

#### Peripheral Blood Monocytes

Circulating monocytes are the primary phagocytic cells and the major APCs in blood (140). Notably, monocytes are able to differentiate into dendritic cells and macrophages for antigen presentation and removal of foreign pathogens, respectively. In humans, peripheral monocytes can be divided into three main subtypes based on the expression of CD14 and CD16 (141): classical monocytes (CD14^++^CD16^-^); intermediate monocytes (CD14^+^CD16^+^) and non-classical monocytes (CD14^-^CD16^++^). Approximately 80% of the total monocytes are classical monocytes, while the non-classical monocytes comprise about 2-11%. Non-classical monocytes retain a highly inflammatory characteristic and their number is elevated in both chronic and acute inflammation. The population of the intermediate monocytes (2-8%) with both inflammatory and phagocytic capacities expands during ZIKA viral infection and is the main target for ZIKA infection during pregnancy (142). Despite the conflicting results on the proportion of classical monocytes in peripheral blood between pregnant and non-pregnant women, classical monocyte number is lower in pregnancy complications such as preeclampsia (143, 144), indicating a possible regulatory role of monocyte in pregnancy.

#### Decidual Macrophages

Decidual macrophages are the second most abundant type of lymphocytes (~20%) and the major antigen-presenting cells (APC) in human decidua during early pregnancy (70). They contribute to maternal-fetal immune homeostasis, spiral artery remodeling and trophoblast functions (145). Decidual macrophages display the transcriptional profile of both classically activated macrophages (M1 macrophages) for immune activation and alternatively activated macrophages (M2 macrophages) with anti-inflammatory and immunosuppressive functions (146, 147). Thus, the decidual macrophages do not fit into the conventional M1/M2 classification of macrophages. Indeed, decidual macrophages show dynamic changes throughout pregnancy (13). For instance, seminal plasma-induced M1 macrophage infiltration contributes to embryo implantation in mice (5) and early placentation (5, 67, 148). As pregnancy proceeds, the M2-dominated microenvironment protects the fetus from rejection. At the time of parturition, M1 macrophage accumulation facilitates uterine contraction (149). The driving forces underlying the phenotype changes remain unclear, yet it is generally believed that the surrounding micro-environment is essential for macrophage transformation and maturation.

Tissue-resident macrophages arise from three subsets of precursors: early yolk sac macrophages, fetal liver monocytes and bone marrow-derived monocytes (150, 151). In other words, the tissue-resident macrophages can be generated by self-renewable macrophages or replenished from circulating monocytes. However, the origin of human decidual macrophages remains uncertain. Kammerer et al. reported a unique CD209^+^CD14^+^CD68^+^ HLA-DR^+^ CD83^-^ proliferating APCs in the decidual of early human pregnancy, suggesting that the human decidual macrophages maintain themselves through self-renewal (152). On the other hand, a gene knockout mice study indicates that the decidual macrophages are replenished by peripheral monocytes expressing circulating lymphocyte antigen 6 complex (Ly6C)^hi^ *via* a Chemokine (C-C Motif) Ligand 2 (CCL2) - CC chemokine receptor-like 2 (CCR2) dependent pathway driven by CSF-1 (153).

#### Effect of pEXO on Circulating Monocytes and Decidual Macrophages

Early pregnancy is in a pro-inflammatory state. Monocytes in the maternal blood are progressively activated in pregnant women compared to non-pregnant women (144). Placenta-derived EVs can transform the phagocytic classical monocytes (CD14^++^CD16^-^) to the intermediate monocytes (CD14^+^CD16^+^) (143) with enhanced migratory capacity and secretion of pro-inflammatory factors such as IL-1β, IL-6, serpinE1, granulocyte-macrophage colony-stimulating factor (GM-CSF), M-CSF and TNF-α (**Supplementary Table 1**) (154, 155). On the other hand, the number of CD14^+^HLA-DR^low^ monocytes is elevated in the maternal blood of the first trimester of pregnancy, and displays an immunosuppressive phenotype when compared with non-pregnant controls (99). Downregulation of HLA-DR endows monocytes with a tolerogenic ability (156). Similarly, tumor-derived exosomes contribute to a systemic immune tolerance *via* modulating the monocyte phenotype. Exosomes from chronic lymphocytic leukemia induce a high expression of PD-L1 in monocytes in a Toll-like receptors 7 (TLR7)-dependent manner (157). Head and neck squamous cell carcinoma-derived exosomes promote monocytes differentiation into an M2 macrophage-like phenotype *via* activation of miR-21 (158).

Studies of pEXO on decidual macrophage are sparse. On the other hand, Nguyen et al. demonstrated that pEXO from pregnant mice are specifically targeted to the lungs and liver, and are taken up by lung interstitial macrophages (97). However, the physiological implications of this observation are unclear. Interestingly, tumor-derived exosomes play a critical role in modulating the differentiation of tumor-associated macrophages (TAMs) *via* exosomal miRNAs, proteins and metabolites (26, 159–161). Similarly, exosomes from the trophoblastic cell line (Swan 71) induce monocyte recruitment and differentiation (**Supplementary table 1**) (155). Another study found that exosome-carrying fibronectin stimulates the production of IL-1β from macrophages (**Supplementary table 1**) (162). Of note, pEXO contain molecules known to promote the induction of decidual macrophages. For example, programmed death-ligand 1 (PD-L1), a factor mainly released by trophoblast in early pregnancy, is identified in trophoblast-derived exosomes, and trophoblast-derived soluble PD-L1 promotes decidual macrophages polarization (163–165). Taken together, pEXO favor pregnancy maintenance by inducing monocyte activation, differentiation and decidual macrophage polarization.

### T Cells

#### Decidual and Peripheral Blood T Cells

T cells are the main cell types responsible for immune surveillance, pathogen recognition and elimination. CD3^+^ T cells constitute ~10% of the decidual lymphocytes in the first trimester. Among them, the CD4^+^ and the CD8^+^ T cells are the two largest groups of T cells accounting for 30-45% and 45-75% of the population respectively (3, 70). During pregnancy, these T cells are immunologically tolerant to the fetus and remain in a constrained cytotoxic phenotype (166). Compared to the circulating CD8^+^ cells, the decidual CD8^+^ T cells are unable to differentiate into the CD8^+^ effector cells as validated by low production of perforin and granzyme B. Moreover, the decidual CD8^+^ T cells show exhausted T cell phenotype with high expression of PD-1, lymphocyte-activation gene 3 (LAG3), cytotoxic T-lymphocyte-associated protein 4 (CTLA4) and T cell immunoglobulin and mucin domain 3 (Tim3) (167). Recent studies further reveal that the CD8^+^ cells are expandable in the decidua with upregulated expression of cell activation markers such as CD25, CD38, CD69 and HLA-DR, as well as enhanced expression of IFN-γ and IL-17A. These partially activated decidual CD8^+^ T cells may be associated with trophoblast invasion and spiral artery remodeling after endothelial monolayer destabilization (126, 168).

Other than the CD8^+^ T cells, CD4^+^ T helper cells (Th) are critical in modulating the immune tolerance to fetal antigens as well. The Th1/Th2 paradigm has been demonstrated to be essential for a successful pregnancy. Furthermore, recent reports have shown that a Th17/Treg balance is well maintained during pregnancy. The number of regulatory Treg cells in both the human decidua and circulation is increased during pregnancy (169–171). Decreased level of CD25^+^Foxp3^+^ Treg is associated with spontaneous abortion (172), preeclampsia (173), and spontaneous preterm birth (174). Furthermore, acute Treg depletion after conception causes embryo resorption along with maternal systemic inflammation and poor endothelial function (92).

Th17 cells are a subset of CD4^+^ T cells presenting a pro-inflammatory phenotype. Although accounting for only ~2% of CD4^+^ T cells, elevated frequency of Th17 cells is related to spontaneous abortion and chorioamnionitis (85, 175–177). Interestingly, the study of Wu et al., showed that Th17 cell numbers in both peripheral blood and decidua are elevated in the first trimester of pregnancy and IL17 could promote trophoblast migration and invasion (82). An inverse relationship of Treg cells and Th17 cells are observed in a wide range of pregnancy complications (81, 85). Thus, the new Th1/Th2/Th17/Treg paradigm indicates that T cell homeostasis is an indispensable factor in pregnancy.

#### Effect of pEXO on T Cells

The roles of pEXO in T cell response have been widely documented. Recent progress suggests that the pEXO mediate immunosuppression *via* transfer of exosomal proteins to the T cells, leading to T cell apoptosis, inhibition of T cell proliferation, induction of Treg differentiation and reduction of T cell cytotoxicity.

##### T Cell Apoptosis

It has long been known that T cell apoptosis in human decidua is a characteristic of early pregnancy. Fas ligand/receptor triggered apoptosis is instrumental in the establishment of immune privilege of the fetus and safeguards its development. pEXO with surface Fas ligand and TNF-related apoptosis-inducing ligand (TRAIL) can induce apoptosis in the Jurkat T cells and activate peripheral blood mononuclear cells (PBMCs) in a dose-dependent manner *in vitro* (42). Moreover, pEXO from maternal blood inhibit T cell activation by down-regulation of CD3 ζ and JAK3, with a more notable effect on CD8^+^ T cells than on CD4^+^ T cells (**Supplementary Table 1**) (178).

##### Treg Differentiation

The role of exosomes in the differentiation of Treg cells has been implicated in tumor immunology (179–181). Tumor-derived exosomes inhibit T cell proliferation, cytotoxic activities and macrophage polarization (179, 182, 183). Exosomes isolated from the normal placenta *via* perfusion also inhibit lymphocyte proliferation and induce Treg/memory T cells differentiation (**Supplementary Table 1**) (184–186). Placental mesenchymal stromal cells (PMSC)-derived exosomes alleviate tubulointerstitial fibrosis by increasing infiltration of the Foxp3^+^/IL17^+^ cells in kidneys of the unilateral ureteral obstruction animal model, indicating the involvement of PMSC-exosomes in Treg differentiation (187). Together, these findings indicate that the pEXO are one of the modulators in Treg differentiation during pregnancy.

##### Cytotoxicity Activity of T Cell

NKG2D ligands such as MHC class I chain-related (MIC) and UL-16 binding protein (ULBP) are expressed on pEXO. Interestingly, the levels of the soluble forms of the MIC protein A and B are negatively correlated with the survival time of cancer patients. The soluble MIC supports tumor escape *via* binding to NKG2D and downregulating its expression on cytotoxic T cells and NK cells. Similarly, pEXO carrying MIC and ULBP down-regulates the expression of NKG2D receptor on CD8^+^ T cells and cytotoxic activities of the CD8^+^ and gamma delta T (γς T) cells (**Supplementary Table 1**) (134). The expression of syncytin-2, an endogenous retroviral protein exclusively expressed on the human placenta, on the pEXO is down-regulated in preeclampsia patients. Lokossou et al. reported that the pEXO bearing syncytin-2 are immunosuppressive *via* reducing Th1 cytokine production in activated PBMCs (**Supplementary Table 1**) (188). Together, these findings indicate that exosomes contribute to immune tolerance through the presentation of MHC molecules or other surface ligands.

## Exosomes in Pregnancy Complications

Pregnancy-associated complications such as preeclampsia, gestational diabetes mellitus and preterm birth, are the major threats to human reproductive health. Despite advances in technology and understanding of pregnancy, the rates of pregnancy-related morbidity and mortality increased slightly over the last two decades (189). The current preventive and prognostic approaches for these complications are limited. Thus, a comprehensive understanding of pregnancy-related complications is much needed for better diagnosis and treatment.

Peripheral blood represents the most widely used biological sample for clinical diagnosis. Circulating fetal DNA in maternal plasma and serum has been used for non-invasive prenatal diagnosis (190). Alterations in pEXO have been demonstrated in pregnancy complications. Thus, pEXO might be a promising alternative for screening the following disorders in pregnancy.

### Preeclampsia

Preeclampsia, characterized by new onset of hypertension and proteinuria, is one of the most severe complications in pregnancy affecting 5% of pregnant women globally (45). Nonetheless, the most effective treatment for preeclampsia is delivery. For decades, its pathogenesis has largely been attributed to 1) compromised trophoblast invasion (191); 2) dysregulated maternal immune tolerance (192) and 3) endothelial dysfunction (193). However, preeclampsia patients often have more than one defect. It is not clear, to what extent, how each of these causes contributed to preeclampsia as a whole. Considering the growing body of evidence that pEXO are key in modulating maternal homeostasis, we hereby summarize these studies to provide new insight on preeclampsia treatment.

pEXO levels in maternal blood of preeclamptic patients are remarkably increased compared to those of normal pregnancy. Moreover, omics data found that the molecular signatures of pEXO are largely different between preeclampsia and normal pregnancy. For example, proteomic analysis of exosomes isolated from maternal plasma by cholera toxin B chain and annexin V binding show that exosomes from preeclamptic patients have a higher expression of serpin peptidase inhibitor (PAI)-1, porphyria cutanea tarda (PCT), S100 calcium-binding protein B (S100b), TGF-β, VEGFR1, natriuretic peptide B (BNP), placental growth factor (PGF) (194, 195). Non-coding RNA-seq of plasma exosomes reveals that miR-486-1-5p and miR-486-2-5p are significant enriched in the preeclampsia group and could be used as potential diagnosis biomarkers (196). More importantly, exosomes from preeclamptic patients elicit preeclamptic symptoms (hypertension and proteinuria) in mice when injected *via* tail veins (197), indicating their indispensable role in preeclampsia occurrence.

Trophoblast invasion and migration are critical in spiral artery remodeling and placentation. In preeclamptic patients, miR-210 is highly enriched in the plasma exosomes compared with that in normal pregnancy, and this in turn contributes to preeclampsia by inhibition of trophoblast invasion through downregulating the potassium channel modulatory factor 1 (198). In addition to the comprised trophoblast function, the miRNA profile is disrupted in preeclampsia exosomes as well. For instance, high levels of miR-517-5p, miR-518b and miR-520h are associated with late-onset preeclampsia (199). Controversially, another study observed that down-regulation of miR-517-5p, miR-520a-5p and miR-525-5p in patients are related to late-onset of preeclampsia (200). The discrepancy might be due to differences in sample preparation, donor ethnicity and gestational age. Given that pEXO only accounts for a small proportion (15-20%) of the total circulating exosomes and remarkable differences in miRNA profiles between pEXO and total plasma exosomes, the data should be interpreted with caution.

Endothelial function is fundamental in modulating blood pressure. Nitric oxide (NO) mediates vasorelaxation *via* an endothelium-dependent pathway. While the diminished activity of endothelial nitric oxide synthase (eNOS), a key enzyme for NO production, is observed in endothelial cells after treatment with preeclamptic pEXO (201). Moreover, preeclamptic patients have a higher level of miR-155 in plasma compared to healthy control and further study showed that it can inhibit eNOS expression in human umbilical vein endothelial cells (HUVEC) (55). An *in vitro* study showed that the macro-EVs from normal pregnancy but not preeclampsia could protect endothelial cells from activation (138). Moreover, an animal study found that human pEXO could relax mesenteric arteries after injection into pregnant mice (202). Another study showed that trophoblast-derived exosomes could promote vascular smooth muscle cell migration (203).

Disrupted maternal immune tolerance is another hallmark of preeclampsia. Syncytin-1/2, which can inhibit T cell activation and proliferation, is reduced in exosomes from preeclamptic patients (204). PD-L1, involved in decidual macrophage polarization and Treg cell differentiation, was found to be remarkably reduced in the placenta and pEXO of preeclamptic patients. Although the proteomic data on pEXO is rare, tissue proteomic results could be an alternative for the exosome study. For example, the expression of neprilysin (NEP), a membrane-bound metalloprotease associated with hypertension, is increased in the preeclamptic placenta at delivery. Interestingly, Manjot et al. recently demonstrated that exosomes from preeclamptic placenta have a higher expression of active NEP when compared to that of in normal placenta (60).

### Gestational Diabetes Mellitus

Gestational diabetes mellitus (GDM) is one of the most common metabolic disorders during pregnancy. It affects ~13.2% of the pregnant mothers in developed countries (205). Without treatment, it may lead to preterm birth, fetal death and other pregnancy complications due to poor placentation induced by hyperglycemia. Although GDM is usually preventable and manageable, infants of mothers with GDM are at increased risk for heart disease, obesity or type 2 diabetes (205–207).

GDM patients have a relatively higher level of total exosomes and pEXO level in maternal plasma (46). Moreover, an *in vitro* study showed that exosomes from GDM patients induced endothelial activation, indicating the importance of pEXO in modulating maternal vascular homeostasis. miRNA compositions in urine-derived exosome and explant culture are different. Exosomes isolated from the urine of GDM patients in the 3rd trimester of gestation have a low level of miR-516-5p, miR-517-3p, miR-518-5p, miR-222-3p and miR-16-5p (208). Those from placental explant culture express another group of miRNAs (miR-125a-3p, miR-99b-5p, miR-197-3p, miR-22-3p and miR-224-5p) (209). Dipeptidyl peptidase IV (DDPIV) modulates glucose hemostasis by cleavage of glucagon-like peptide 1 (GLP-1) and DDPIV inhibitors are used for type 2 diabetes treatment. Manu Vatish *et al.* found that exosomes isolated from GDM placenta through perfusion had an upregulation of DDPIV by 8-fold (210). Moreover, exosomes from GDM pregnancy remarkably reduce migration and glucose uptake of skeletal muscle cells (209). Similarly, plasma exosomes from GDM patients also induce glucose intolerance, reduce glucose-induced insulin secretion and cause poor insulin responsiveness in mouse model (211).

GDM may arise from metabolic dysregulation of adipose tissue, which is critical in the modulation of insulin sensitivity (212). In general, normal pregnancy is accompanied by increased total adipose mass. Maternal body mass index (BMI) has a strong association with the risk of GDM, indicating excessive adipocytes are a potential stressor for placentation (213). Adiponectin and leptin, mainly produced by the placenta during pregnancy, have a wide range of functions in adipose tissue such as vascularization, adipocyte enlargement and expansion (207). Exosomes from adipose tissue of GDM patients altered placental glucose metabolism by increasing gene expression of the glycolysis and gluconeogenesis pathways (214). Thus, pEXO may participate in maternal metabolism *via* modulating the activity of adipose tissue.

### Preterm Birth

Preterm birth, also known as premature birth, generally refers to birth at less than 37 weeks of gestational age (215). Nowadays, preterm birth is the leading cause of perinatal morbidity and mortality and has a long-term effect on the health of the fetus (216). For instance, premature infants are vulnerable to heart defects, cognitive disabilities, and respiratory illnesses (217). Nonetheless, the cause of preterm birth is still unclear.

Studies on pEXO of preterm birth are rare. Unlike preeclampsia and GDM, the level of pEXO in preterm birth is significantly decreased compared to full-term pregnancy (218). Placenta senescence and fetal membrane inflammation are generally believed to be the causes of preterm birth. Proteomic study of exosomes from preterm plasma indicates that alterations in protein composition are associated with inflammatory and metabolic signals (219). A similar result was found in amniotic fluid-derived exosomes of preterm patients (220). In animals, Plasma exosomes of CD-1 mice from late-gestation (E18), not early-gestation (E9), induce preterm labor in mice, indicating that exosomes might function as one trigger in labor initiation (221). Moreover, the exosomal miRNA profile of maternal plasma is different between mothers delivering at term and preterm (222, 223). A comprehensive analysis of the exosomal miRNAs reveals that the miRNAs target genes are associated with TGF-β signaling, p53, and glucocorticoid receptor signaling (222). Despite the inconsistency and irreproducibility of miRNA sequencing results, exosomal miRNAs are still suggested as an alternative approach for the diagnosis of preterm birth. Together, these studies indicate that pEXO participate in the processes of labor and delivery.

## Discussion

Studies of exosome had been tremendously increased in the last two decades and exosomes are gradually demonstrated to be a perfect tool for drug delivery. However, our understanding of exosome biogenesis and the underlying forces that navigate them to their destination is still lacking.

Currently, most studies on pEXO are conducted *in vitro* due to ethical constraints in regard to manipulation of the maternal-feto-placental unit and lack of proper animal models. pEXO isolated from placenta tissue at mid (Abortion)- or term (Delivery)-gestation, may not represent it’s *in vivo* functions at early pregnancy. Therefore, the content and biological activities of pEXO at different gestation periods should be investigated. Furthermore, the alternation of pEXO signatures observed from late gestational samples in clinical studies would possibly be the consequence rather than the cause of the pregnancy complications. A large prospective study of the first trimester pEXO isolated from plasma/placenta tissue from pregnant women who develop pregnancy complications at late gestation should be carried out. Apart from that, *in vitro* manipulations (Such as perfusion and explant culture) during exosome isolation may disrupt the molecular signature of pEXO. Thus, pEXO isolated by different isolation methods should be compared in order to establish a standard isolation technique and to set a standard parameter for diagnostic purposes.

**Figure 3:**
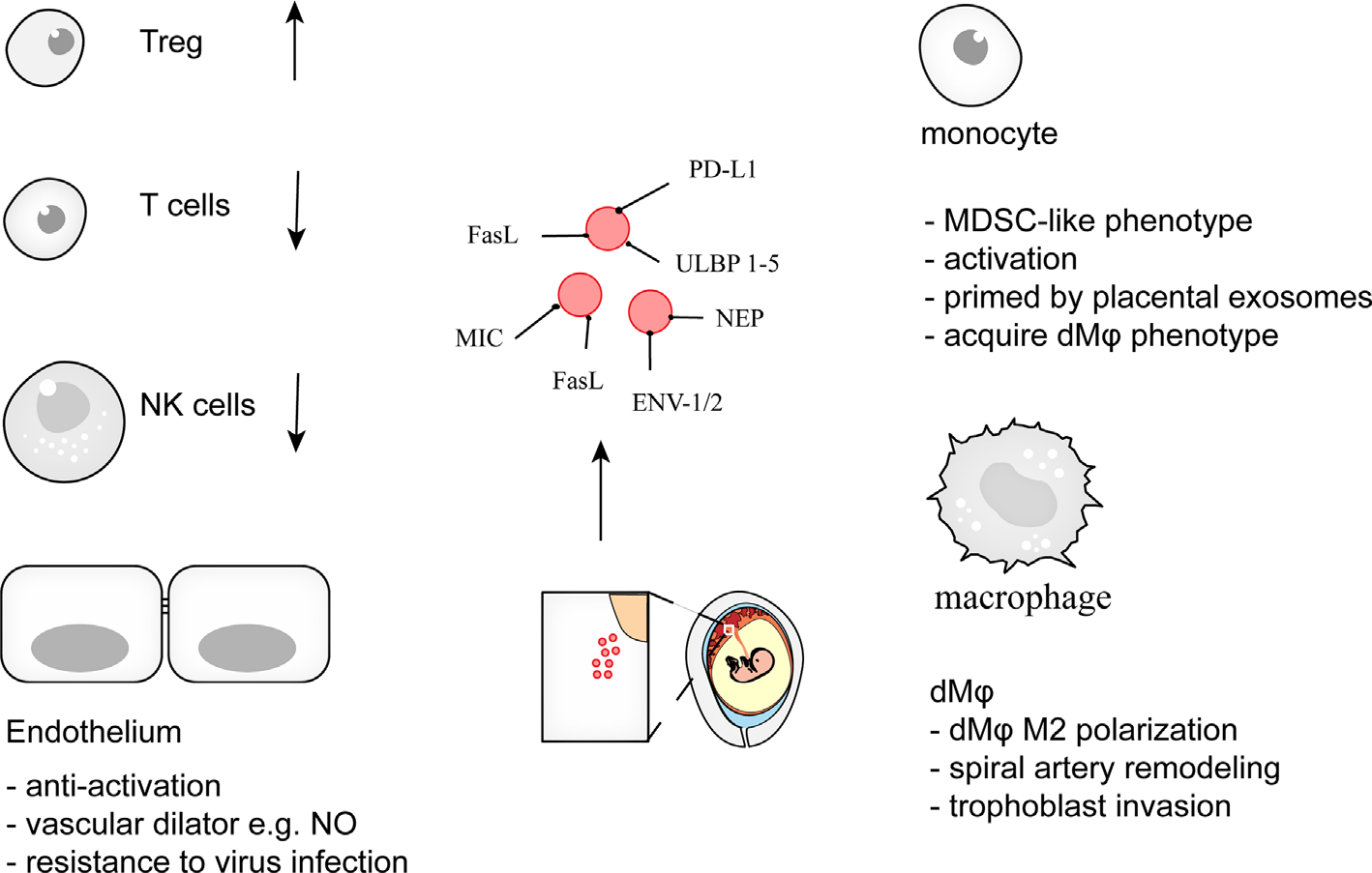
pEXO contribute to maternal tolerance toward the fetus during pregnancy. Exosomes from placenta, syncytiotrophoblast (STB) in particular, support pregnancy *via* induction of Treg differentiation, restraint of cytotoxic activities of T cells and NK cells, promotion of decidual macrophage polarization, and endowing endothelial cells with viral resistance. Disruption of maternal immune tolerance is associated with adverse pregnancy complications such as miscarriage, preeclampsia. The specific cargoes within the pEXO represent the potential target for prenatal diagnosis and pregnancy-related disease screening.

In summary, pregnancy is a complex physiological process with a wide range of systemic adaptations in the mother’s body. The placenta, the frontline of the maternal-fetal interface, makes these happened in a coordinated way. Exosome, as a signal carrier, links the mother and the fetus and is a key player in immune cell activation, differentiation, maturation and endovascular homeostasis (**Figure 3**). Thus, advances in pEXO research will deepen our understanding of pregnancy and may provide new insight on the prevention and treatment of pregnancy-related complications.

## Author Contributions 

Conceptualization – PC, C-LL and KB. Writing – original draft preparation, KB. Writing – review and editing, XL, JZ, C-LL, PC, EN and WY. Illustration – XL and KB. Supervision – C-LL and PC. Funding acquisition – C-LL and EN. All authors contributed to the article and approved the submitted version.

## Funding

This work was supported in part by the Hong Kong Research Grant Council Grant (17115619 and 17118917), National Natural Science Foundation of China (81971396) and Sanming Project of Medicine in Shenzhen.

## Conflict of Interest

The authors declare that the research was conducted in the absence of any commercial or financial relationships that could be construed as a potential conflict of interest.
