# Supplementary material for: Placenta-Derived Exosomes as a Modulator in Maternal Immune Tolerance During Pregnancy
Source: Front Immunol. 2021 May 11;12:671093. doi: 10.3389/fimmu.2021.671093 (PMC8144714; doi:10.3389/fimmu.2021.671093)
Supplement: Supplementary file 1 [file Table_1.docx]

Supplementary table 1. Summary of the effect of exosomes on NK cells, monocytes/macrophages and T cells during pregnancy.

| **Target Cell** | **Phase** | **Exosome Source** | **Purification Method** | **Main Findings** | **Ref** |
| --- | --- | --- | --- | --- | --- |
| NK cell | 1 st trimester | Explant Culture | Ultracentrifugation | Exosomes downregulated the in vitro cytotoxicity through its cargo UL-16 binding proteins (ULBP) by decreasing the expression of NKG2D in NK cells. | [135] |
|  | 1 st trimester | Maternal Serum | Total Exosome Isolation Reagent (Thermo) | Exosome-bounded B7H6, one of NKG2D ligand, impaired NK cytotoxicity. | [138] |
|  | 1 st trimester | Maternal Serum | Exoquicktm Solution (System Biosciences, Inc.) | Induce NK apoptosis | [134] |
| Monocyte | Swan-71  cell line | Conditioned Medium | Ultracentrifugation | Trophoblast-derived exosomes recruited and educate monocytes and induce pro-inflammation and chemokine production. | [156,157] |
| Macrophage | Swan-71  cell line | Conditioned Medium | Ultracentrifugation | Exosome-bound fibronectin induced macrophage IL1β production | [164] |
| T cell | 1 st trimester | Explant Culture | Ultracentrifugation | Exosomes downregulated the in vitro cytotoxicity through its cargo UL-16 binding proteins (ULBP) and MIC by decreasing the expression of NKG2D in T cells. | [135] |
|  | 1 st trimester | Explant Culture | Ultracentrifugation | Fasl and TRAIL-carrying placental exosomes induce Jurkat T cell and PBMC apoptosis | [42] |
|  | Labor | Maternal Blood | Chromatographic Isolation | Placenta-derived exosomes from blood of term women suppress T cell activation by inhibiting CD3-zeta expression | [180] |
|  | Term placenta | Perfusion | Ultracentrifugation | Induce T cell differentiate to T regulatory cells Treg and memory T cells | [186,187,226] |
|  | Term placenta | Primary Cell | Perfusion | Placenta-derived exosomes reduce Th1 cytokine production in activated PBMC | [190] |
